# Determination of the Deacetylation Degree of Chitooligosaccharides

**DOI:** 10.3390/md15110332

**Published:** 2017-10-25

**Authors:** Yao Jiang, Chuhan Fu, Sihui Wu, Guihua Liu, Jiao Guo, Zhengquan Su

**Affiliations:** 1 Guangdong Engineering Research Center of Natural Products and New Drugs, Guangdong Pharmaceutical University, Guangzhou 510006, China; jiangyaoabcd@163.com (Y.J.); chuhanfu@163.com (C.F.); 2 Guangdong Metabolic Diseases Research Center of Integrated Chinese and Western Medicine, Key Unit of Modulating Liver to Treat Hyperlipemia SATCM (State Administration of Traditional Chinese Medicine), Guangdong Pharmaceutical University, Guangzhou 510006, China; 3 Guangdong Food and Drug Vocational Technical School, Guangzhou 510663, China; wsh2709@163.com; 4 Shenzhen Center for Disease Control and Prevention, 8 Longyuan Road, Nanshan District, Shenzhen 518055, China; gliu_686@hotmail.com

**Keywords:** chitooligosaccharides, deacetylation, bromocresol green, acid-base titration, first-order derivative UV spectra, ^1^H NMR

## Abstract

The methods for determination of chitosan content recommended in the Chinese Pharmacopoeia and the European Pharmacopoeia are not applicable for evaluation of the extent of deacetylation (deacetylation degree, DD) in chitooligosaccharides (COS). This study explores two different methods for assessment of DD in COS having relatively high and low molecular weights: an acid-base titration with bromocresol green indicator and a first order derivative UV spectrophotometric method for assessment of DD in COS. The accuracy of both methods as a function of molecular weight was also investigated and compared to results obtained using ^1^H NMR spectroscopy. Our study demonstrates two simple, fast, widely adaptable, highly precise, accurate, and inexpensive methods for the effective determination of DD in COS, which have the potential for widespread commercial applications in developing country.

## 1. Introduction

Chitins originate from crustaceans such as shrimp, crab, insects, mushrooms, green algae, and silkworms. They are one of the most abundant renewable biopolymers on earth and can be obtained inexpensively from marine sources with more than 10 GT on an annual basis [1]. The molecular structure of chitins consists of *N*-acetylated glucosamine (GlcNAc) and 2-amino-2-d-glucose (d-glucosamine, GlcN), and chitin contains a high proportion of GlcNAc [2]. However, their poor solubility limits usage of this natural resource. One possible means of addressing this challenge involves chitosan, which is the product of chitin deacetylation under alkaline conditions. Chitosan is a linear polysaccharide containing various proportions of GlcN [3]. It has applications in nutritional [4], agricultural [5], and medical areas [6,7,8]. However, its poor solubility in aqueous solutions and its high viscosity are problematic for those applications.

**Figure 1:**
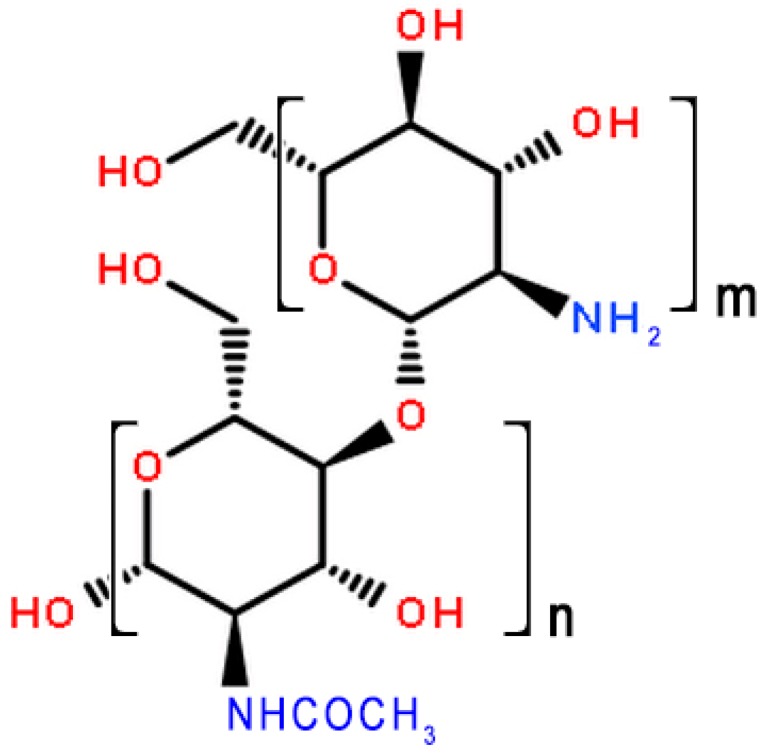
The molecular structures of COS (*n* = 2–20), COS: Chitooligosaccharides.

Chitooligosaccharides (COS) are hydrolyzed products of chitosan, and previous studies indicate that they may be produced from chitosan using either chemical and physical methods, as well as enzymatic hydrolysis [9,10]. COS contains 2–20 glucosamine residues which are attached through β-d-(1-4) glycoside linkages, similar to chitin and chitosan (Figure 1). The family of COS compounds have received much attention because of their small molecular weight, good aqueous solubility, and diverse biological activity [11]. Applications of interest include antibacterial efficacy [12], antitumor activity [13], moderation of cholesterol levels [14,15,16], and weight loss [17,18].

The degree of deacetylation (DD, %) is defined as the molar fraction of GlcN in the copolymers (chitosan) composed of GlcNAc and GlcN [19]. The DD value of a COS sample is one of the most important factor in assessing its applications in the medical, nutritional, sewage treatment, and biotechnological fields [20]. It can also be related back to the specific biological and structural properties and functions of chitin or chitosan, and it should be clear that chitosan is the deacetylated form of chitin and it must be characterized by a degree of acetylation when a degree of deacetylation is valid for chitin, the initial form of the polymer nearly fully acetylated.

For chitin, chitosan, and COS, measurement of the DD is crucial in determination of their chemical structures, physical properties, and interrelation. It is also essential in providing a basis from which the functions and applications can be predicted and optimized [2,21]. Therefore, a suitable method of obtaining reliable DD values is essential for diverse researchers working in those fields.

To date, several techniques have been developed and applied for determination of DD in chitosan [19,20,22,23,24,25,26,27,28,29,30,31,32,33,34]. These have different levels of precision and accuracy. The currently accepted methods are specific to the concerned organization: U.S. Pharmacopoeia uses ^1^H NMR spectroscopy [35], *European Pharmacopoeia* has approved the first-order derivative UV spectrophotometric method [36], and Chinese Pharmacopoeia prefers an acid-base titrametric procedure using methyl orange as the indicator [37]. However, a standard method for determination of DD in COS for use by researchers, companies, and even state organizations is still lacking. Arguably, this predicament has resulted from variation in chitosan properties.

For COS, a method for DD determination based on ^1^H NMR spectroscopy has been reported [26,29,32]; however, its large expense limits its application in developing countries. Unfortunately, the alternative procedures for DD determination in chitosan, which have been endorsed by the Chinese Pharmacopoeia and European Pharmacopoeia (vide supra), are highly inaccurate for evaluation of the DD in COS.

Our research team has long been engaged in the study of COS [38] and has developed COS products such as the COS tablet [39] and the COS capsule [40]. We found that the weight loss function of COS is equal to that of Orlistat, the only Over the Counter (OTC) weight loss pill currently on the market, and that its side effects are significantly lower than those of Orlistat [18]. The DD value of a COS sample is the one of the most important factor in assessing its quality, and the methods for determination of chitosan content recommended in the Chinese Pharmacopoeia and the European Pharmacopoeia are not applicable for evaluation of the extent of DD in COS. A need therefore exists for a method for DD determination in COS which is easy to perform, inexpensive, widely adaptable, and highly accurate. Our research group has addressed this challenge by pioneering the development of two new methods which are applicable to COS having several different molecular weight averages, and herein we report the results of those investigations for DD determination in COS using: (a) the acid-base titration with bromocresol green indicator; and (b) the first-order derivative of a UV spectrometric trace. The parameters we selected to optimize for each method were the indicator p*K*_a_ and color change (titrametric method) and the solvent (UV method). Furthermore, the accuracy of both new methods in samples having relatively high and low molecular weights (COS_A_ and COS_B_) were assessed, and those results, through comparison to those from the ^1^H NMR spectroscopic method, were verified.

## 2. Results and Discussion

### 2.1. ^1^H NMR Spectroscopy

**Figure 2:**
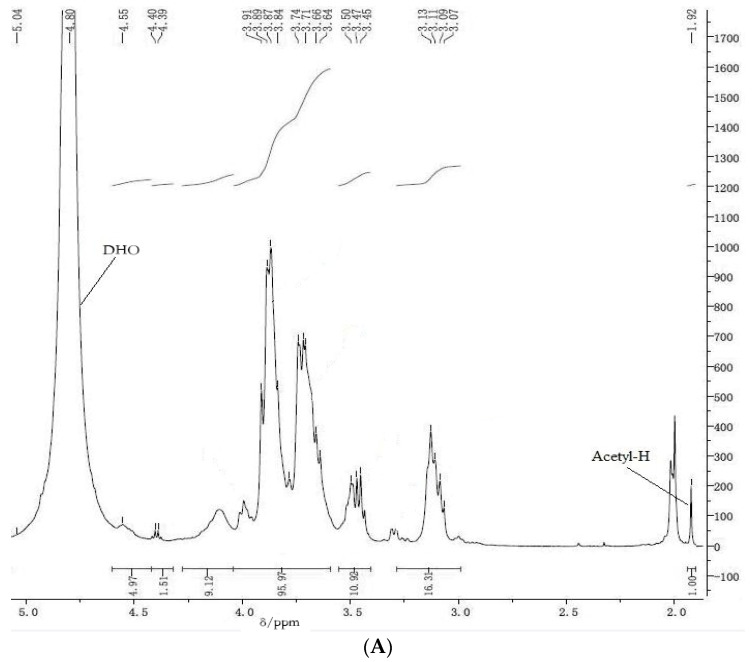

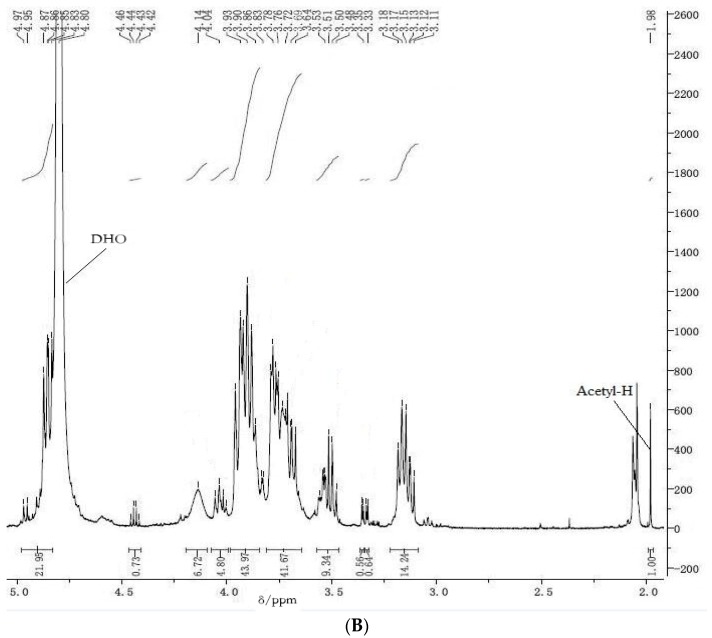
(**A**) ^1^H NMR spectrum (500 MHz,) of solutions of COS_A_ in D_2_O; and (**B**) ^1^H NMR spectrum (500 MHz) of solutions of COS_B_ in D_2_O.

The ^1^H NMR spectra of COS_A_ and COS_B_ are shown in Figure 2A,B, respectively. Those figures also provide information on chemical shift assignments, integration values, and relative numbers for those protons. Functional groups containing –OH or –NH_2_ moieties and with ^1^H NMR shifts near the H_2_O resonance at 4.80 ppm were identified through H/D exchange in deuterium oxide.

Averaged ^1^H NMR spectroscopic shifts of various protons in COS from the literature were collected and assembled (Table 1). The values at ca. δ 1.92 ppm are assigned to CH3 of the *N*-acetyl group in GlcNAc. After normalizing the intensity of that shift, the remaining resonances from the ring positions, which occur at ca. δ 2.6–4.2 ppm, were summed and substituted as *A*_2_ into Equation (1), to calculate DD values for COS_A_ and COS_B_ (Table 2); values of 93.55 ± 0.15% and 92.85 ± 0.13% (*n* = 3), respectively, were obtained.

### 2.2. First-Order Derivative UV Spectrophotometry

#### 2.2.1. Selection of Solvent and Maximum Wavelength

**Figure 3:**
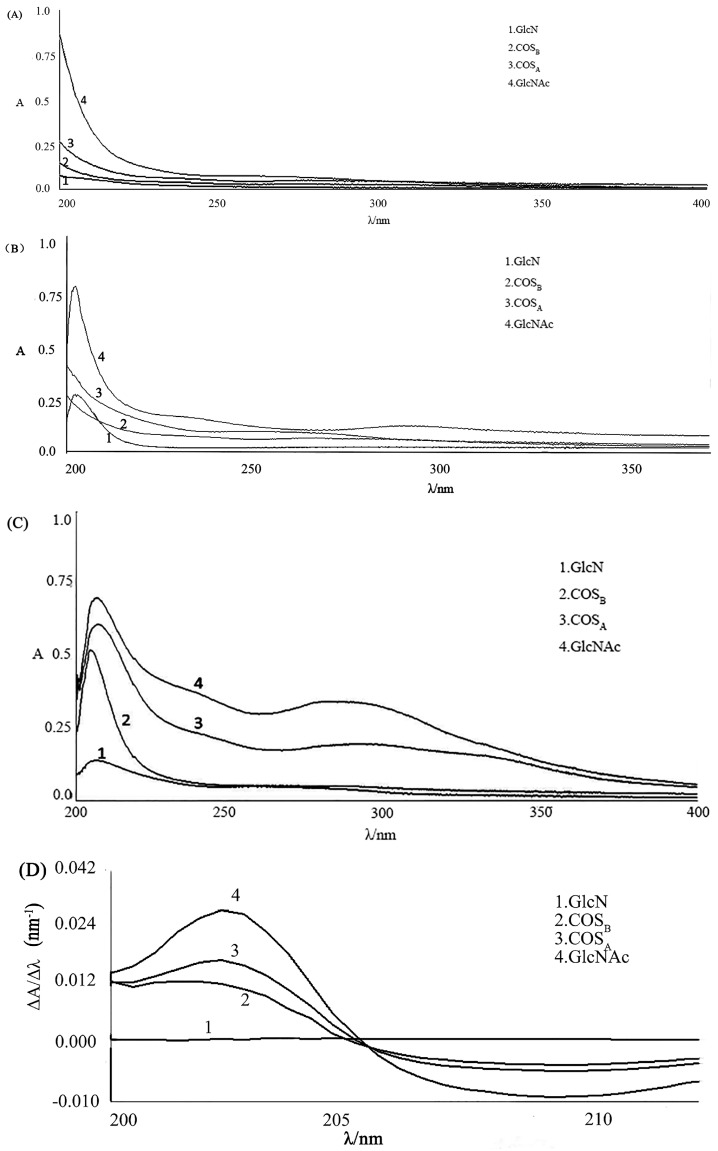
Comparison of zero-order UV spectra of different species of interest in this study, recorded in: water (**A**); 0.3 M acetic acid solution (**B**); and 0.3 M hydrochloric acid solution (**C**). Comparison of first-order derivative UV spectra of different species of interest in this study, recorded in 0.3 M hydrochloric acid solution (**D**).

Using water as the solvent, we observed no λ_max_ in the zero-order UV spectra of the GlcN, GlcNAc, COS_A_, and COS_B_ analyte solutions (Figure 3A). Using acetic acid solution as the solvent, we observed no λ_max_ in the zero-order UV spectra of the COS_A_, and COS_B_ analyte solutions (Figure 3B). This contrasts the spectra recorded in HCl solution as the solvent, which gave λ_max_ = 204 nm (Figure 3C). We conclude that 0.3 M HCl solution is the most appropriate solvent for measurement of the simple UV spectra. Under the best condition, the analyte solution of GlcN, GlcNAc, COS_A_, and COS_B_ absorbance under the model of first-order derivative over the 200–215 nm range was recorded (Figure 3D), which shows that first-order derivative UV spectra can interfere in the elimination of GlcN for determining the DD of COS_A_ and COS_B_.

#### 2.2.2. Linearity of First-Order Derivative UV Spectra

**Figure 4:**
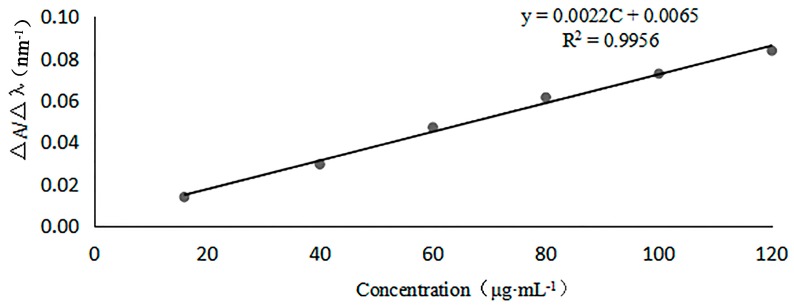
Calibration curve from first-order derivative UV spectra of GlcNAc (16.0–120.0 μg/mL) in 0.3 M hydrochloric acid solution.

First-order derivative absorption spectra of standardized GlcNAc solutions were recorded under the dominant wavelength of 204 nm, a baseline wavelength of 202 nm of the quantitative model, and the amplitude ∆*A*/∆λ (∆*A* = *A*_204nm_ − *A*_202nm_, ∆λ = 2 nm) of GlcNAc. The amplitude values (∆*A*/∆λ) and six standardized GlcNAc solutions were used to construct the calibration curves for first-order derivative UV spectra (see Figure 4). The regression equation was ∆*A*/∆λ = 0.0022*C* + 0.0065 (*C*, μg/mL), the correlation coefficient *R*^2^ = 0.9956 (*n* = 6), the linear range was 16–120 μg/mL, the limit of detection (LOD) was 2.0 μg/mL, and the limit of quantification (LOQ) 6.5 was μg/mL. Those results indicate a good sensitivity of first-order derivative UV spectra. We anticipate that this procedure will be a valuable tool for the determination the DD of COS.

#### 2.2.3. Accuracy of First-Order Derivative UV Spectra

The results showed that the recovery values are greater than 98% with low standard deviation, indicating high accuracy of the proposed analytical methods. The corresponding results were calculated according to Equation (2), and are summarized in Table 3. The average recovery rates, 98.1 ± 0.63% (*n* = 3) and 98.4 ± 0.62% (*n* = 3) for COS_A_ and COS_B_, respectively, were very good.

#### 2.2.4. Precision of First-Order Derivative UV Spectra

Precision studies revealed %RSD values within acceptable limits (<3%), reflecting the precision and robustness of the method within the selected range. The DD of those samples was calculated according to Equation (2) and within-day RSD and between-day RSD are summarized in Table 4. We can thereby see that the first-order derivative UV spectroscopic method has excellent precision under the current experimental conditions. Furthermore, the results are consistent with those from ^1^H NMR spectroscopy, and indicate that molecular weight is not a factor in the determination of the DD of COS using the first-order derivative UV spectroscopic method.

### 2.3. Moisture Determination

Moisture is a very important factor for the determination of DD in COS by acid-base titrimetric method, and, in this research, moisture values were 6.0% and 5.8% for COS_A_ and COS_B_, respectively.

### 2.4. Acid-Base Titration with Methyl Orange Indicator

There is an experimental error of ca. 60% for the values of COS_A_ and COS_B_ in the determination of DD in COS according to the acid-base titrimetric method used in the Chinese Pharmacopoeia with methyl orange indicator (2–3 drops of 1% M methyl orange indicator solution in water). Those values were 30.37 ± 0.70% and 30.30 ± 0.57% (*n* = 6) for COS_A_ and COS_B_ (data not shown), respectively. The colors for both the COS_A_ and COS_B_ solutions were pale yellow, which was close to the color observed near to the end point for the titrametric determination using the methyl orange indicator. We consider this a possible explanation for the poor experimental accuracy of this method for DD determination in COS.

### 2.5. Acid-Base Titration with Bromocresol Green Indictor

#### 2.5.1. Effect of pH

The GlcN in COS exists mainly in the form of its hydrochloride salt, resulting in a pH of approximately 5.12 for aqueous solutions of COS. The pH value of a COS solution is the most important factor during the titrametric COS titration procedure. Consequently, to carry out an accurate titration, it is first necessary to completely liberate the amino functionality through an appropriate adjustment of the pH of the aqueous COS solution. Our study shows that the determination of DD in COS over the COS_A_-COS_B_ range and the bromocresol green indicator method works best at pH 8.0 according to Table 5. This finding is consistent with the results of our ^1^H NMR spectroscopic investigations. It is important to note that for pH < 8, incomplete liberation of GlcN in COS occurs, and that this will lead to overestimation of the mass of GlcN which is thereby found. Therefore, the conditions for solubilization and analysis need to know under which form was isolated of COS: in excess of HCl, it is the directly water soluble chlorhydrate form, and, in neutral conditions, it is the –NH_2_ form, and the polymer is insoluble in neutral and alkaline conditions.

#### 2.5.2. Selection of Indicator

The DD value is one of the most important factors in assessing chitosan and its derivative applications in the medical, nutritional, and biotechnological fields. In that context, the acid-base titrametric method, using methyl orange as the indicator, is currently accepted [37]. However, methyl orange is inappropriate at COS concentrations above those in our proposed method; indeed, we have observed a measurement error of ca. 60%. One reason for this limitation lies in the pale yellow color of aqueous solutions of COS. On the other hand, the bromocresol green indicator possesses a p*K*a of 4.9. Under acidic and basic conditions, this indicator displays pale yellow and turquoise colors, respectively. It also has p*K*a of 4.9, hence its effectiveness is in the pH 3.8–5.4 range. We believe that these properties make bromocresol green the best indicator for the COS titration end point.

#### 2.5.3. Stability and Repeatability of Acid-Base Titration Using Bromocresol Green Indicator

To evaluate the applicability of the indicator titration method, we have examined its stability using samples of COS having two different molecular weights COS_A_ and COS_B_ under the best condition. The working solutions of COS_A_ and COS_B_ were analyzed by acid-base titration using bromocresol green indicator methods at 0, 2, 4, 6, 12, and 24 h after preparation. The behavior of COS_A_ and COS_B_ remained unchanged during the entire period of study (data not shown). In order to assess the repeatability of the bromocresol green titrametric method for DD determination in COS, the procedure was repeated six times for each of the samples of COS_A_ and COS_B_. DD values of 93.32 ± 0.27% and 92.59 ± 0.53%, respectively, were thereby obtained (Table 6 and Table 7). It should be noted that those results indicate that molecular weight does not appear to be a factor in applying the bromocresol green method for DD determination in COS.

### 2.6. Comparison of Different Methods for DD Determination

The data in Table 8 indicate that the first-order derivative UV spectroscopic procedure and the bromocresol green acid-base titration indicator method agree with the results obtained using the ^1^H NMR spectroscopic method. The U.S. Pharmacopoeia accepts the ^1^H NMR spectroscopic method for determination of DD in chitosan, and the Europe Pharmacopoeia prefers the first-order derivative UV spectroscopic procedure. On the other hand, Chinese Pharmacopoeia has endorsed the methyl orange indicator titrametric method. However, a broadly accepted method is still lacking for researchers in industry and government for determination of DD in COS. This has resulted from chitosan samples obtained from different sources having different compositions. While the ^1^H NMR spectroscopic method can be applied to the determination of DD in COS, its expense leads to it being less attractive in developing countries.

## 3. Materials and Methods

### 3.1. Materials

Commercial samples of COS used in this study were obtained from Qingdao (Shangdong, China) and had average molecular weights of 1000 Da and 3000 Da, respectively. The DD of the commercial COS were >90% in glucosamine hydrochloride. The *N*-acetylglucosamine used in this study was purchased from Sigma Aldrich (St. Louis, MO, USA) and had a purity greater than 99%. The deuterium oxide used had an isotopic purity of 99.8%, and was obtained from Shanghai Macklin Biochemical Co., Ltd. (Tianjin, China). Bromocresol green and methyl orange were purchased from Tianjin Damao Chemical Reagent Factory (Tianjin, China) and methyl orange from Tianjin Zhiyuan Chemical Reagent Co., Ltd. (Tianjin, China), respectively. Sodium hydroxide pellets (NaOH), hydrochloric acid (HCl), and potassium hydroxide pellets were obtained from Tianjin Kermel Chemical Reagent Co., Ltd. (Tianjin, China). All other reagents and solvents were of analytical grade and used without further purification. All water used in the extraction and analysis was distilled and deionized.

### 3.2. ^1^H NMR Spectroscopy

The following procedure, from the U.S. Pharmacopoeia, was used for ^1^H NMR spectroscopic determination of chitosan content. Approximately 20–30 mg COS samples were dissolved in 1 mL deuteroxide. Aliquots of 0.5–1.0 mL of the sample solution were transferred to a standard 8-mm NMR spinning tube. A spectral window of 8012 Hz, 297 K, and an acquisition time of 4.08 s were used for all measurements. ^1^H NMR spectra were recorded on a Bruker 500 MHz spectrometer (Bruker, Billerica, MA, USA) at 297 K. The quantitation method involved scanning the concerned sample over δ 0–5 ppm; every sample was evaluated three times. The average area of the segment in the δ 3–6 ppm range was recorded as *A*_1_, and represented the seven protons adjacent to the oxygen atoms within the sugar molecule ring structure. The average areas below the resonance traces from the CH_3_ protons on the acetyl groups appeared at ca. δ 2 ppm and were recorded as *A*_2_ according to the equations listed below. The ^1^H NMR spectrum of a typical COS sample shows resonances at ca. 1.9–2.1 ppm from CH_3_ of the *N*-acetyl group in GlcNAc. The shifts of the protons attached to positions C_2_–C_6_ on the sugar ring appear at ca. 2.6–4.2 ppm [4]. The DD of COS samples was determined according to Equation (1) [35]:

DD(%) = [1 − (6 × *A*_2_/3 × *A*_1_)] × 100, (1)

where *A*_1_ are the protons integral values of positions C_2_–C_6_ on the sugar ring and *A*_2_ are the protons integral values of the three *N*-acetyl protons of GlcNAc.

### 3.3. Determination of DD in COS Using the First-Order Derivative of UV Spectra

The UV spectra method of analysis is widely used in the analysis of drugs in pharmaceutical formulations due to its good sensitivity and cost effectiveness. Over the last three decades, derivative spectrophotometry has been extensively used in the determination of drugs in multicomponents having overlapping spectra, which eliminates interference from the formulation matrix by using the zero-crossing techniques. For GlcNAc, the absorption maximum occurs at λ_max_ = 204 nm in 0.3 M hydrochloric acid solution. GlcN also has an absorption which interferes with the determination of GlcNAc at 204 nm. However, the first-order derivative UV spectroscopic method compensates for this phenomenon. The procedure described in the European Pharmacopoeia for determination of chitosan DD in COS, which uses a first-order derivative UV spectroscopic method, is described as follows. According to this method, ca. 0.1 g of accurately weighed samples of COS are dried overnight at 45 °C at 1 atm pressure. The samples are then dissolved in 50.0 mL 0.3 M HCl solution with vigorous stirring. For analysis, aliquots of 1.0 mL of this solution are diluted to 10.0 mL with hydrochloric acid solution. As necessary, further dilutions of the analyte solution are performed. In this study, the maximum wavelength for analyzed solutions was obtained at 200–400 nm at a slit width of 2 nm, a scanning speed of 20 nm·min^−1^, a time constant of 4 s, and a chart speed of 10 cm·min^−1^. Cuvettes with 10 mm path length were used in the UV spectrophotometer (Presee T10CS, Beijing, China). Then, the analyte solution absorbance under the model of spectrum scanning over the 200–215 nm range was recorded, and the absorbance was transformed into differential calculation at a time. The absorbance of GlcNAc in the COS was determined by measuring the first derivative signal at 200–215 nm. All measurements were blanked against the same HCl solution. The masses of GlcNAc in the COS analyte solutions were determined according to the calibration curve. The DD was calculated according to Equation (2) [36]:

(2) $$\mathrm{DD}(\%)=\frac{C_{\mathrm{sample}}-C}{C_{\mathrm{sample}}-\frac{42}{203}C}\times 100,$$

where *C*_sample_ is the concentration of COS in the analyte solution (μg/mL); *C* is the concentration of GlcNAc in the analyte solution, as determined from the calibration curve (μg/mL); 203 is the molecular mass of GlcNAc fragment in COS polymer, according to C_8_H_13_NO_5_; and 42 is the difference in molecular masses between the GlcNAc and GlcN fragments.

#### 3.3.1. Linearity of First-Order Derivative UV Spectra

For linearity, six different standard solutions of GlcNAc, having concentrations of 16.0, 32.0, 64.0, 80.0, 100.0, and 120.0 μg/mL, were prepared in 0.3 M HCl solution. The first derivative of the UV absorption curve at λ = 200–215 nm of each solution was measured, and absorbance of GlcNAc under the dominant wavelength of 204 nm and a baseline wavelength of 202 nm were recorded so that amplitude ∆*A*/∆*λ* (∆*A* = *A*_204nm_ − *A*_202nm_, ∆*λ* = 2 nm) and GlcNAc concentrations were obtained. Then, a calibration curve was made. The LOD and LOQ were determined by 3.3 *σ*/*s* and 10 *σ*/*s* criteria, respectively, where *σ* is the standard deviation of the analytical signal and *s* is the slope of the corresponding calibration curve.

#### 3.3.2. Accuracy of First-Order Derivative UV Spectra

Accuracy of the first-order derivative UV spectroscopy was determined by calculating the recoveries of GlcNAc by the standard addition method, in which pre-analyzed samples (100 μg/mL) were taken, and GlcNAc was added at three different levels, i.e., 80%, 100%, and 120%. The total amount of GlcNAc was estimated by using the proposed methods in triplicate for every level. The percent recovery of the added GlcNAc was calculated as percent recovery = (*D*t − *D*s)/*D*a) × 100, where *D*t is the total sample concentration measured after standard addition; *D*s is the sample concentration in the formulation mixture; *D*a is the sample concentration added.

#### 3.3.3. Precision of First-Order Derivative UV Spectra

To evaluate the first-order derivative UV spectra method precision, different concentrations were prepared from sample stock solution and analyzed three times within the same day and on three separate days to evaluate within-day and between-day precision, respectively. Under the first-order derivative model, the amplitude ∆*A*/∆λ (∆*A* = *A*_204nm_ − *A*_202nm_, ∆λ = 2 nm) of COS samples is examined, and the equivalent amount of GlcNAc for the COS samples is analyzed through the calibration curve. The DD was calculated according to Equation (2). From the obtained data, the percent relative standard deviation (% RSD) was calculated.

### 3.4. Acid-Base Titrametric Determination of DD in COS Using Methyl Orange Indicator

The procedure described in the Chinese Pharmacopoeia for determination of chitosan DD in COS, using acid-base titration and methyl orange indictor, was followed. According to this method, ca. 0.5 g of accurately weighed samples of COS were dried overnight at 45 °C at 1 atm pressure. Afterwards, the samples were dissolved in 32.0 mL distilled and deionized water by stirring with a gradient mixer (TH-500A, Shanghai Chemical Co. Ltd., Shanghai, China). To the resulting solution, 18.00 mL of 0.3 M aqueous HCl solution (standardized to 0.314 M) was added. The volume of added aqueous HCl solution was noted as *V*_HCl_, followed by addition of 2–3 drops of 1% M methyl orange indictor. Afterwards, the solution was back-titrated with 0.15 M aqueous NaOH (standardized to 0.146 M), until transition of the color from red to yellow had occurred. The volume of added standard aqueous NaOH solution was noted as *V*_NaOH_. Calculation of DD was according to Equation (3) as follows [37]:

(3) $$\mathrm{DD}(\%)=\frac{(C_{\mathrm{HCl}}\times V_{\mathrm{HCl}}-C_{\mathrm{NaOH}}\times V_{\mathrm{NaOH}})\times 0.016\times 100\%}{G\times (100-W)\times 9.94\%},$$

where *C*_HCl_ is the concentration of standardized aqueous HCl solution (M); *C*_NaOH_ is the concentration of standardized aqueous NaOH solution (M); *V*_HCl_ is the volume of standardized aqueous HCl solution (mL); *V*_NaOH_ is the volume of standard NaOH aqueous solution (mL); *G* is the weight of COS sample (g); *W* is the aqueous concentration of COS sample (%); 0.016 is the equivalent weight of amino groups corresponding to 1 mL 0.1 M HCl (g); and 9.94% is the theoretical equivalent mass of amino groups, expressed as percentage.

### 3.5. Acid-Base Titrametric Determination of DD in COS Using Bromocresol Green Indicator

According to this procedure, ca. 0.5 g of accurately weighed samples of COS were dried overnight at 45 °C at 1 atm pressure. The samples were dissolved in 32.0 mL distilled and deionized water by stirring with a gradient mixer. The pH of the COS solutions were adjusted to 8.0 and monitored using a pH meter. An 18.00 mL aliquot of 0.3 M aqueous HCl solution (standardized to 0.314 M) was added to the solution. The volume of the aqueous HCl solution added was noted as *V*_HCl_, and then 2–3 drops of 1% bromocresol green indicator solution in ethyl alcohol were added. Afterwards, and while stirring, the solution was back-titrated with 0.15 M (standardized to 0.146 M) aqueous sodium hydroxide solution until transition of the indicator color from yellow to blue-green had occurred. The volume of added standardized NaOH aqueous solution was noted as *V*_NaOH_. DD was calculated according to Equation (3).

### 3.6. Moisture Determination

For determination of moisture levels, ca. 0.3–0.5 g sample of COS was weighed on the sample plate of an infrared moisture determination instrument (Sartorius, M7, Berlin, Germany). The sample was shaken for homogeneous distribution across the plate, moisture was determined until the scale heating test no longer moved, and the results were recorded.

## 4. Conclusions

In conclusion, the determination of DD for COS was critically assessed using two new analytical procedures. One was an acid-base titrametric method that uses bromocresol green indicator, and the other employed the first-order derivatives of UV spectra. We investigated the sensitivity, accuracy, and precision of the DD values obtained by these methods. The influence of molecular weight on both methods was also of concern. These two new procedures are simple, easy to perform, and inexpensive under a variety of circumstances within industry, government, and academia. As COS is a new natural resource having many applications throughout society, it is anticipated that these two new methods will be widely used and accepted in developing countries.

## Figures and Tables

**Table 1:** Averaged ^1^H NMR shift values (δ, D_2_O, 25 °C) of various protons in COS.

Residue	Proton			
	H_1_	H_2_	H_2–6_	Acetyl-H
GlcNAc	4.55–4.65	-	2.6–4.2	1.92
GlcN	5.15	3.1–3.2	2.6–4.2	-

**Table 2:** Results of measured the DD (COS_A_, COS_B_) using ^1^H NMR spectrum (*n* = 3).

Sample	*A*_1_	*A*_2_	DD (%)	$\bar{\mathrm{DD}}$ (%)	RSD (%)
COS_A_-1	30.30	1.00	93.40	93.55	0.15
COS_A_-2	31.06		93.56		
COS_A_-3	31.70		93.69		
COS_B_-1	27.55	1.00	92.74	92.85	0.13
COS_B_-2	28.50		92.98		
COS_B_-3	27.89		92.83		

*A*_1_: integral values of protons on positions C_2_–C_6_ on the sugar ring; *A*_2_: integral values of protons on the *N*-acetyl methyl group of GlcNAc); DD: deacetylation degree; RSD: relative standard deviation.

**Table 3:** Recovery of first-order derivative UV spectra (*n* = 3).

Sample *	Added (μg/mL)	Test (μg/mL)	Recovery (%)	Average Recovery (%)	RSD (%)
COS_A_	20.0	19.6 ± 0.29	98.5 ± 1.5	98.1	0.63
COS_A_	40.0	38.9 ± 0.32	97.3 ± 1.2		
COS_A_	60.0	59.2 ± 0.25	98.7 ± 1.2		
COS_B_	20.0	19.5 ± 0.27	97.5 ± 1.7	98.4	0.62
COS_B_	40.0	39.6 ± 0.34	99.0 ± 1.9		
COS_B_	60.0	58.9 ± 0.28	98.1 ± 1.7		

* Concentration of COS_A_ and COS_B_ in the pre-analyzed samples is 100 μg/mL.

**Table 4:** Precision of first-order derivative UV spectra (*n* = 3).

Sample	*C* (μg/mL)	DD (%)	$\bar{\mathrm{DD}}$ (%)	RSD (%)	Within-Day RSD (%)	Between-Day RSD (%)
COS_A_-1	40.2	93.4	93.4	0.35	0.81	0.95
COS_A_-2	39.8	93.0			0.83	0.93
COS_A_-3	40.5	93.8			0.82	0.90
COS_B_-1	38.5	92.8	92.6	0.33	0.77	0.92
COS_B_-2	38.9	92.8			0.84	0.90
COS_B_-3	39.1	92.2			0.79	0.95

**Table 5:** Results of DD (COS_A_, COS_B_) measurements at different pH using acid-base titration with bromocresol green indicator (*n* = 3).

pH	7.60	7.80	7.90	8.00	8.10	8.20
COS_A_ (%)	87.45 ± 0.68	90.94 ± 0.61	91.64 ± 0.66	93.44 ± 0.73	95.62 ± 0.80	97.65 ± 0.75
COS_B_ (%)	88.14 ± 0.79	89.86 ± 0.80	90.82 ± 0.76	92.69 ± 0.81	94.25 ± 0.78	97.12 ± 0.83

**Table 6:** Volumes of aqueous hydrochloric acid solution, sodium hydroxide solution, and calculated deacetylation degree values obtained for COS_A_ samples (*n* = 6).

Sample *	*G* (g)	*V*_HCl_	*V*_NaOH_	DD (%)	$\bar{\mathrm{DD}}$ (%)	RSD (%)
1-1	0.50040	18.00	20.02	93.34	93.32	0.27
1-2	0.50022	18.00	20.10	92.98		
1-3	0.50010	18.00	20.05	93.25		
1-4	0.50021	18.00	19.95	93.73		
1-5	0.50035	18.00	20.05	93.20		
1-6	0.50027	18.00	20.00	93.44		

*G*: weight of COS_A_ sample; *V*_HCl_: volume of standard HCl aqueous solution (mL); *V*_NaOH_: volume of standard NaOH aqueous solution (mL); * Moisture value of analyzed samples is 6.0% for COS_A_.

**Table 7:** Volumes of aqueous hydrochloric acid solution, sodium hydroxide solution, and calculated deacetylation degree values obtained for COS_B_ samples (*n* = 6).

Sample *	*G* (g)	*V*_HCl_	*V*_NaOH_	DD (%)	$\bar{\mathrm{DD}}$ (%)	RSD (%)
1-1	0.50018	18.00	20.00	93.28	92.59	0.53
1-2	0.50022	18.00	20.21	92.23		
1-3	0.50038	18.00	20.25	92.00		
1-4	0.50001	18.00	20.05	93.07		
1-5	0.50051	18.00	20.15	92.48		
1-6	0.50042	18.00	20.15	92.49		

*G*: weight of COS_B_ sample; *V*_HCl_: volume of standard aqueous HCl solution (mL); *V*_NaOH_: volume of standard NaOH aqueous solution (mL); * Moisture value of analyzed samples is 5.8% for COS_B_.

**Table 8:** Results of DD (COS_A_ and COS_B_) measurements by two new methods (*n* = 3).

Sample	^1^H NMR Spectrum (%)	First-Order Derivative of UV Spectra (%)	Acid-Base Titration with Bromocresol Green Indicator (%)
COS_A_	93.55 ± 0.15	93.4 ± 0.35	93.19 ± 0.70
COS_B_	92.85 ± 0.13	92.6 ± 0.33	92.50 ± 0.73

## Abbreviations

COS	chitosan oligosaccharides
MW	molecular weight
COS_A_	average molecular weight ≤1000 Da of chitosan oligosaccharides
COS_B_	average molecular weight ≤3000 Da of chitosan oligosaccharides
DD	degree of deacetylation
GlcN	2-amino-2-d-glucose (d-glucosamine)
GlcNAc	2-acetamido-2-d-glucose
^1^H NMR	nuclear magnetic resonance hydrogen spectrum
UV	ultraviolet
